# Cardiac Ameliorative Effect of *Moringa oleifera* Leaf Extract in High-Fat Diet-Induced Obesity in Rat Model

**DOI:** 10.1155/2020/6583603

**Published:** 2020-02-27

**Authors:** Lamia Mabrouki, Ilhem Rjeibi, Jihen Taleb, Lazhar Zourgui

**Affiliations:** ^1^Research Unit of Active Biomolecules Valorisation, Higher Institute of Applied Biology of Medenine (ISBAM), University of Gabes, 4119 Medenine, Tunisia; ^2^Faculty of Sciences of Gafsa, 2112 Gafsa, Tunisia

## Abstract

The consumption of a high-fat diet is linked to the development of obesity and considered a risk factor for cardiovascular diseases. The aim of this study was to evaluate the effect of the methanolic extract of *Moringa oleifera* leaves (MEML) on the high-fat diet- (HFD-) induced obesity and cardiac damage in rats. MEML, at a dose of 200 mg/kg/bw and 400 mg/kg/bw, was orally administrated to obese rats for 12 weeks. *M*. *oleifera* leaves were proved to be rich in nutrients and minerals. Diversity of phenolic compounds in MEML was evidenced via LC-ESI-MS analysis. The chronic administration of HFD in rats led to an increase in the body weight gain, total cholesterol, and triglycerides and reduction in the HDL-C levels. The obtained results indicated a significant increase (*p* < 0.05) in the cardiac marker enzyme level in obese rats. A significant decrease (*p* < 0.05) in the levels of cardiac catalase (CAT), glutathione peroxidase (GPx), and superoxide dismutase (SOD) activities was accompanied with an increase of malondialdehyde (MDA) level in the high-fat diet group when compared to those of the control. The treatment with the MEML alleviated abnormalities in the serum biochemical parameters, balanced the antioxidant status, and reestablished the normal histological structure of the heart especially in the case of the higher concentration. *Moringa oleifera* leaves may be a promising candidate in the management of obesity and its related complications such as heart problems.

## 1. Introduction

Obesity is a common disease whose prevalence is associated with a combination of genetic, nutritional, and environmental factors. It has now become among the most growing public health issues. A 2016 World Health Organization (WHO) survey on the globally increasing prevalence of obesity reported that 1.9 billion adults were overweight, more than 600 million of which obese [1]. Obesity has been proven to increase various health risks such as cardiovascular disease, diabetes mellitus, fatty liver disease, cancers, and asthma [2]. The strong association between obesity and cardiovascular disease is an area of intensive research. A growing body of evidence suggests that increasing abdominal adiposity is associated with a greater risk of developing heart failure [3].

The existing antiobesity medications have been effectively implemented in regulated weight-loss treatments. However, they pose risk severe adverse effects which outweigh their advantageous properties. Most recent studies on the treatment of obesity and its related comorbidities have focused on the potential role of different plant preparations that can exert a positive effect on the mechanisms involved in this pathology [4]. These potential health benefits may be attributed to macronutrients, micronutrients, and secondary metabolites present in plants.

*Moringa oleifera*, Lam (Moringaceae), is one of the Brassica aboriginal vegetables, native to the Indian subcontinent and commonly found in tropical and subtropical countries [5]. For centuries, people around the world have used different parts of this plant (roots, leaves, and seeds) for numerous therapeutic applications in the treatment of inflammation and infectious diseases, along with cardiovascular, gastrointestinal, hematological, and respiratory disorders [6, 7]. Recent trials revealed that *M*. *oleifera* leaves might contribute to prevent obesity as well as obesity-related complications [8, 9]. The present study evaluates the therapeutic potential of *Moringa oleifera* leaf extract in the treatment of obesity as well as its protective effect on the cardiac disorders induced by a high-fat diet feeding.

## 2. Material and Methods

### 2.1. Chemicals

Gallic acid, Folin-Ciocalteu reagent, catechin, rutin, thiobarbituric acid, catalase (CAT), and malondialdehyde (MDA) were purchased from Sigma–Aldrich Co. (Sigma, St. Louis, USA). Standards (iron (Fe), zinc (Zn), calcium (Ca), potassium (K), magnesium (Mg), manganese (Mn), phosphorus (P), and sodium (Na)) were obtained from Merck (Darmstadt, Germany). Superoxide dismutase (SOD) and glutathione peroxidase (GPx) assay kits were purchased from Randox Laboratory Ltd. Methanol was purchased from PanReac (Barcelona, Spain). All other chemicals and solvents were of analytical grade and were obtained from Sigma–Aldrich (Sigma, St. Louis, USA).

### 2.2. Plant Collection

Fresh leaves of *M*. *oleifera* were collected from the Tataouine region in south Tunisia, in August 2017. The plant was identified by a botanist, Dr. Boulbaba Ltayef (the Faculty of Sciences of Gabes, Tunisia), and a voucher specimen (MO-0317) was deposited in the Herbarium of the Faculty of Sciences, University of Gafsa, Tunisia. The plant leaves were cleaned, shade dried at room temperature, ground into fine powder, and stored at −20°C until use.

### 2.3. Physicochemical Composition of *M*. *oleifera* Leaves

Moisture content of the leaf powder was determined according to the AOAC [10] method by oven drying the sample at 110°C until constant weight was attained. Similarly, ash content was determined according to the AOAC [10] method and the value was obtained by incineration of the sample at 550°C in a muffle furnace until a constant weight. Nitrogen estimation was carried out using the micro-Kjeldahl method with some modifications [11]. Crude proteins were subsequently calculated by multiplying the nitrogen content by 6.25. Ether extracts were determined by means of a Soxhlet extractor, using n-hexane as a solvent. Crude fibers were estimated by acid-base digestion with 1.25% H_2_SO_4_ (*w*/*v*) and 1.25% NaOH (*w*/*v*) solutions [12]. The carbohydrate content was calculated by removing from 100% the amount of moisture, total fat, protein, and ash [10]. The total soluble sugars were measured using phenol-sulfuric according to the method of Al-Harrasi et al. [13]. Pure glucose was used as the standard. The energy value estimation was done by summing values for crude proteins, crude fats, and carbohydrates, respectively, multiplied by the factors (2.44, 8.37, and 3.57), as proposed by Martin and Coolidge [14]. Mineral composition was obtained by applying the method of Rjeibi et al. [15].

### 2.4. Preparation of *M*. *oleifera* Leaf Extract

The fine powder of *M*. *oleifera* leaf was extracted with methanol (10 g/200 mL) for 24 h at room temperature and under magnetic stirring (magnetic stirrer, Heidolph, Germany). The extract was centrifuged at 4,500 rpm for 25 min, then filtrated with Whatman Millipore filter paper (0.45 *μ*m) and concentrated on a rotary evaporator (40°C water bath temperature) (EYELA, Tokyo, Japan). Finally, the resulting extract was stored in the dark at 4°C till use.

### 2.5. Total Phenolic and Flavonoid Contents

The total phenolic content was estimated using the Folin-Ciocalteu method [16]. A calibration curve was obtained using gallic acid as a standard. Total phenolic content was expressed as mg gallic acid equivalent (GAE)/g dry extract. The total flavonoid content was determined by aluminum chloride colorimetric assay [17], and rutin was used as a standard. The results were expressed as mg rutin equivalent (RE)/g dry extract. Tannin content in the *M*. *oleifera* extract was determined using the method described by Chupin et al. [18]. A standard curve was prepared with catechin, and the results were expressed as mg catechin equivalent (CE)/g dry extract.

### 2.6. Liquid Chromatography-Electrospray Ionization-Tandem Mass Spectrometry (LC-ESI-MS) Analysis of Phenolic Compounds

A Shimadzu LC-20ADXR pump with a SIL-20AXR autosampler (40°C) was used for HPLC analysis. The injection volume was 5 *μ*L, and the separation temperature was 75°C in a Discovery BIO Wide Pore C18 (250 mm × 4 mm, 5 *μ*m) column. The flow rate was fixed at 0.4 mL/min, and the mobile phase pooled with eluent A (5% methanol, 95% H_2_O, and 0.15% acetic acid) and eluent B (50% acetonitrile, 50% water, and 0.15% acetic acid). The eluent flow rates were as follows: 0-14 min from 10 to 20% eluent B, 14-27 min with 20% eluent B, 27-37 min from 20 to 55% eluent B, 37-45 min with 55% eluent B, and 45-52 min from 55 to 100% eluent B. A Shimadzu UFLC XR–2020 single quadrupole mass spectrometer equipped with an electrospray interface (ESI: electrospray ionization) was used for MS analysis. ESI conditions were as follows: an electrospray source (source block temperature, 450°C; desolvation temperature, 280°C; capillary voltage, 2.7 kV; cone voltage, 35 V), a nebulizing gas flow 1.5 L/min, and a drying gas flow 15 L/min. Identification of compound was carried out by comparison with standards.

### 2.7. Animal Studies

#### 2.7.1. Animals

In this study, three-month-old healthy male Wistar rats (*n* = 24), with about 120-140 g body weight, obtained from the Central Pharmacy of Tunis, Tunisia, were kept for a two-week adaptation period under controlled conditions of temperature (22 ± 2°C), relative humidity (50% ± 4%), and a constant photoperiod (12 h light/dark cycle). Food and water were provided *ad libitum*. The rats were treated in accordance with the Tunisian approved code of practice in compliance with the European Convention for the Protection of Vertebrate Animals used for Experimental and other Scientific Purposes (Council of Europe No. 123, Strasbourg, 1985).

#### 2.7.2. Induction of Obesity

During the study period, two diets were used: The normal control group was given a normal diet (ND) and the control group was maintained on HFD as illustrated in Table 1 [19]. HFD was prepared by mixing all the ingredients and baking in an oven at 40°C for 24 hours.

When the Lee index value of rats fed with HFD was above 0.3, the model was considered to be successfully established [20].

#### 2.7.3. Treatment

The rats were randomly divided into four groups of six each. Treatment groups were defined as follows:

Group 1 (C): control rats fed with normal diet

Group 2 (HFD): rats fed with a high-fat diet

Group 3 (HFD+MEML 200): rats fed with a high-fat diet and received MEML at 200 mg/kg.bw by oral gavage

Group 4 (HFD+MEML 400): rats fed with a high-fat diet and treated by gavage with MEML at 400 mg/kg.bw.

Animals did not show any clinical signs of toxicity with the maximum dose of 2,000 mg/kg bw [21]. Rats were dosed by oral gavage at doses of 200 and 400 mg/kg.bw in a volume of 1.5 mL/kg distilled water.

During the experiment, the body weight gained and feed intakes were reported at weekly intervals. The feed efficiency ratio was calculated using the equation: (total weight gain/total feed intake) × 100. At the end of the experiment, all the animals were rapidly sacrificed by decapitation under ether inhalation anesthesia to minimize handling stress. Blood serum was obtained by centrifugation (1,500 rpm, 15 min, 4°C), and the hearts were removed, cleaned of fat, and frozen at -80°C for biochemical and histological studies.

### 2.8. Biochemical Estimations

The serum levels of total cholesterol (TC), triglyceride (TG), high-density lipoprotein-cholesterol (HDL-C), creatine kinase-MB (CK-MB), aspartate transaminase (AST), and alanine transaminase (ALT) were evaluated using commercial kits (Spinreact, Girona, Spain) on an automatic biochemical analyser (Vitalab Flexor E, Spankeren, Netherlands) in the biochemical laboratory of the Regional Hospital of Gafsa, Tunisia. Serum low-density lipoprotein cholesterol (LDL-C) values were estimated using the Friedewald equation [22]:

(1) $$\begin{array}{c} \text{LDL}‐\text{C}=\left[\text{TC}-\left(\text{HDL}‐\text{C}+\frac{\text{TG}}{5}\right)\right]. \end{array}$$

### 2.9. Assessment of Antioxidant Parameters and Lipid Peroxidation in Heart Tissue

One gram of rat hearts were cut into small pieces and homogenized in 2 mL of tris buffer solution (TBS) (pH 7.4) using an Ultra-Turax homogenizer; then, the mixtures were centrifuged (5,000 rpm, 30 min, 4°C). The supernatants were collected and used to determine (1) the level of lipid peroxidation by measuring the thiobarbituric acid reactive substances (TBARS) according to Yagi's method [23]; (2) the total superoxide dismutase (SOD) activity, following the method of Misra and Fridovich [24]; (3) the glutathione peroxidase (GPx) activity, using the method of Flohe and Gunzler [25]; and (4) the catalase (CAT) activity according to the method of Aebi [26]. The total sulfhydryl groups (TSH) were evaluated at 412 nm after reaction with 5,5′-dithiobis-(2-nitrobenzoic acid) [27].

### 2.10. Histopathological Analysis

Small pieces of rat heart tissues were immersed in a fixative solution (4% formaldehyde in phosphate buffer, pH 7.6), dehydrated in ethanol, and washed with toluene. The paraffin embedded tissue blocks were made 5 mm thick sections using a rotary microtome and dried overnight in an oven at 37°C. The sections were later stained with hematoxylin and eosin (H&E). Tissue preparations were observed and microphotographed under a light BH2 Olympus microscope.

### 2.11. Statistical Analysis

All results were presented as mean ± standard deviation (SD). Statistical significance was determined using one-way analysis of variance (ANOVA) followed by a Tukey post hoc test. *p* < 0.05 was considered statistically significant. Pearson correlation coefficients (*r*) were used for the estimation of relationships between variables.

## 3. Results

### 3.1. Chemical Composition of *M*. *oleifera* Leaves

Table 2 presents the proximate composition of *M*. *oleifera* leaves on a dry weight basis. The sample was found to contain moisture (6.82%) and ash (10.73%). Crude protein had a value of 25.87%, while ether extract and fiber amounted to 7.28% and 7.98%, respectively. Carbohydrates and sugar content amounted to 41.32% and 9.18%, respectively. The total energy value of 100 g of dry leaves was 350 kcal. The mineral element concentration of the *M*. *oleifera* plant leaf powder is also presented in Table 2. Calcium was determined to be the most abundant of all the macroelements analysed herein, followed by K> Na > Mg. Mean contents of the microminerals Fe, Mn, Zn, and Cu were 34.56, 4.72, 2.93, and 0.86, respectively.

### 3.2. Phenolic, Flavonoid, and Condensed Tannin Contents

The results summarized in Table 3 indicate that *M*. *oleifera* leaves possessed a high content of total phenolic (247.53 mg gallic acid equivalents/g extract) and flavonoids (34.13 mg/g mg rutin equivalents/g extract). An appreciable amount of tannins was also detected in the sample extract (10.5 mg catechin equivalents/g extract).

### 3.3. HPLC-ESI-MS Analysis

Using 31 authentic standards, we noticed that the LC-ESI-MS analysis of the methanol leaf extract revealed 18 phenolic compounds in which flavonoids were the most concentrated group. Hyperoside (316,822 *μ*g/g extract) and quercetrin (204,685 *μ*g/g extract) were reported as primary compounds (Table 4).

### 3.4. Body Weight, Feed Intake, and Feed Efficiency Ratio

Consumption of HFD for 12 weeks caused a significant (*p* < 0.05) increase in body weight gain in the HFD group when compared to the control group (Table 5). The percentage of body weight gain in the rats, fed the HFD supplemented with both low and high MEML doses, was significantly lower (*p* < 0.05) than that in the HFD group; in the case of low- and high-dose MEML, the body weight gain percentage reached 145.33% and 126.15%, respectively.

Concerning feed intake, a slight change was detected between the HFD group and the control group or MEML-treated groups. Feed efficiency ratio was significantly increased in the HFD group, as compared to the control group. The oral administration of MEML resulted in a significant decrease (*p* < 0.05) in feed efficiency ratio levels; the higher dose exhibited a more substantial result than that of the lower dose of the extract (Table 5).

### 3.5. Assays of Serum Markers

As shown in Table 5, the rats fed with HFD for 12 weeks exhibited a significant increase (*p* < 0.05) in serum TC (95.76 ± 11.09 mg/dL), TG (265.15 ± 7.12 mg/dL), and LDL-C (59.33 ± 0.16 mg/dL) levels when compared with the control group. However, these rises were significantly (*p* < 0.01) attenuated by supplementation with different MEML concentrations. Additionally, MEML markedly moderated the serum HDL-C level which had been significantly decreased (*p* < 0.05) in the HFD rats.

The effects of MEML supplementation on the levels of serum cardiac marker enzyme CK-MB, AST, and ALT are shown in Table 5. In this study, CK-MB, AST, and ALT amounts increased significantly in HDF-treated rats (*p* < 0.01). Administration with MEML to HDF-treated rats resulted in a significant reduction (*p* < 0.05) in the levels of CK-MB, AST, and ALT enzymes when compared with the HFD group. The high-dose MEML supplementation (400 mg/kg) appeared to be more effective in enzyme-lowering levels.

### 3.6. Antioxidative Activities

To determine whether obesity is a primary factor contributing to oxidative stress in the heart tissue, we analysed lipid peroxidation, a marker of oxidative damage. Lipid peroxidation expressed by thiobarbituric acid reactive substances (TBARS) significantly increased (*p* < 0.05) in the HFD-treated group compared to the control group. However, administration of *M*. *oleifera* extract resulted in a significant (*p* < 0.01) reduction of these TBARS to almost normal values (0.77 ± 0.3 nmol/mg protein). Levels of glutathione peroxidase (GPx), catalase (CAT), and superoxide dismutase (SOD) in the hearts of both control and experimental rats are illustrated in Table 6. A significant decrease (*p* < 0.05) in the activities of these antioxidant enzymes were perceived in animals which received a high-fat diet when compared with the control group. As shown in Table 6, antioxidant enzyme levels were significantly, and dose-dependently, enhanced by administration with *M*. *oleifera* extract. Moreover, a significant (*p* < 0.01) decrease in cardiac TSH value was detected in the HFD-treated rats as compared with the control group. In contrast, a considerable improvement in the level of this nonenzymatic antioxidant was observed in the MEML-treated rats when compared with the HFD rats.

### 3.7. Histopathological Examination

**Figure 1:**
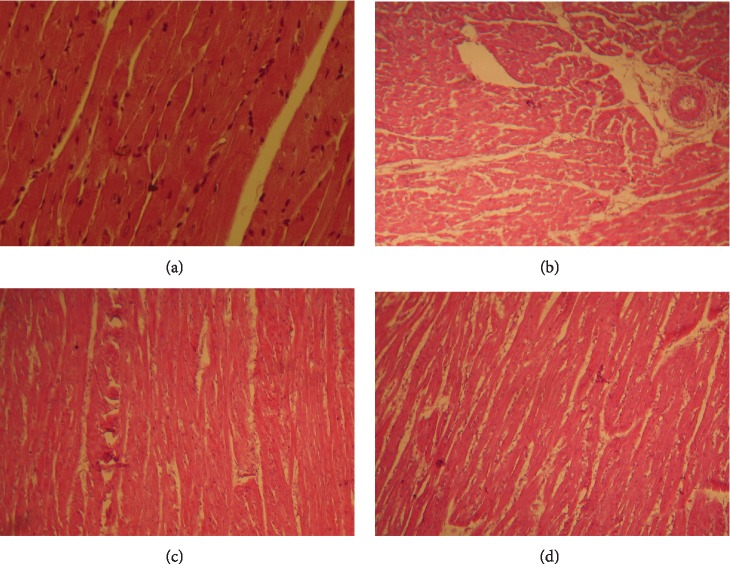
Representative micrographs from the heart exhibiting the protective effect of methanol *M*. *oleifera* leaf extract (MEML) on high-fat diet-induced cardiac injury in rats. (a) Control groups showing normal cardiac architecture. (b) High-fat diet-treated group showing anarchized myocardial fibers associated with interstitial edema and inflammatory cellule collections. (c) High-fat diet group received MEML (200 mg/kg) showing repair in the histological sections. (d) High-fat diet group treated with MEML (400 mg/kg) showing normal structure almost similar to control. Heart sections were stained using hematoxylin-eosin method. Magnifications: ×400.

Microscopic observation of the heart section in control rats showed a normal cardiac histological architecture with regularly arranged cardiac myofibers and muscle bundles (Figure 1(a)). In contrast, the HFD-treated rats revealed damaged heart sections evidenced by disorganized myocardial fibers associated with interstitial edema, inflammatory cell collections, and myocardial fibrosis (Figure 1(b)). However, the histopathological abnormalities were not to the same extent of severity in the MEML-treated animals; the heart revealed soft inflammatory mononuclear collections and absence of necrosis areas (Figure 1(c)). The rats treated with 400 mg/kg MEML exhibited a model of myocardial fiber arrangement similar to that of the control group (Figure 1(d)).

### 3.8. Pearson's Correlation Study

Correlation analyses were performed by using Pearson's correlation test to clarify the relation between different parameters. Feed intake, body weight gain, and feed efficiency ratio showed a strong positive correlation with total cholesterol, AST, ALT, and TBARS because the correlation coefficients (*r*) were all greater than 0.93 (*p* < 0.001) (Table 7). The body weight gain and feed efficiency ratio were also positively and strongly correlated with CK-MB (*r* = 0.941 and 0.960, respectively; *p* < 0.001).

## 4. Discussion

The study showed that *M*. *oleifera* leaves are an interesting source of nutrients. The leaves contained an appreciable amount of crude protein (25.87 g/100 g DW). The high protein value recorded in this study suggests that the leaves can be ranked as a potential source of plant protein and, therefore, could be used as a protein supplement in diet. The obtained results of ether extract analysis (7.28 g/100 g DW) are in agreement with those reported in the literature, ranging from 2.23 to 8% [28, 29]. Available carbohydrate content was moderate, which is consistent with data reported by Aslam et al. [30] (38.21 g/100 g DW). It is worth noting that the levels of fat and carbohydrate of the leaf sample are of particular nutritional significance as they may intervene in the body's energy requirements without causing overweight due to the low caloric value of the leaves. *M*. *oleifera* is an interesting source of fiber (7.98 g/100 g DW) that helps to maintain a healthy digestive system and produces a positive adjustment in serum cholesterol levels as well as a measurable reduction in the peak level of serum glucose [31]. Ash content is the reflection of the mineral preserved in the sample. The reported value of ash (10.73 g/100 g DW) indicated that the leaves were a good source of minerals, fairly higher than the range of 5.43 to 5.75 g/100 g DW reported for some edible woody plants [32]. The predominant macroelement in the leaf sample was Ca. Calcium which plays a vital role in the maintenance of bones, thus preventing osteoporosis [33]. High level of potassium was revealed in our sample, accounting for 1,134.12 mg/100 g DW. Potassium is required in the body for maintaining reasonable sodium-potassium ratio assisting the prevention of heart disease and stroke [34]. Iron was the major microelement detected in our sample (34.56 mg/100 g DW). The highly nutritious value of *M*. *oleifera* leaves enables its use as a nutraceutical and panacea for many chronic diseases [35]. Moreover, the plant leaves can play a role in monitoring lipid metabolism as well as the prevention of obesity in animal models and even in humans.

Phytochemicals has gained recent interest due to their perceived role in protecting against metabolic diseases. In this study, we found that *M*. *oleifera* extract was rich in total phenolic content (247.53 mg gallic acid equivalents/g extract), flavonoids (34.13 mg/g mg rutin equivalents/g extract), and tannins (10.5 mg catechin equivalents/g extract). The obtained result revealed a total phenolic value far greater than that recorded in previous studies [36, 37] reporting the following data for phenolics: 96.30 ± 0.28 mg GAE/g and 48.35 ± 0.05 mg GAE/g of extract, respectively. A considerable body of literature highlights the contribution of oxidative stress and phenolic compounds to, respectively, the pathogenesis and prevention of human diseases [38]. Further, many authors found a high correlation between phenolic compounds and antioxidant activities [39]. Current evidence has proven that phenolics and flavonoids are more efficient antioxidants than vitamins C or E and carotenoids [40]. Hence, plants which contain great amounts of phenolic compounds are believed to exert health-beneficial effects by counteracting oxidative stress, thus reducing the risk of chronic diseases.

In order to identity the different active compounds of our plant leaves, we employed LC-ESI-MS profile analysis. The value of the application of LC-ESI-MS on plant biochemical research stems from its ability to simultaneously monitor retention time of the isolated compound peak and determine accurate molecular weight which is often a characteristic of that particular compound [41]. Results demonstrated that the *M*. *oleifera* extract was rich in phenolic acids, especially flavonoids (Table 4).

Using MS analysis, a total of 26 different flavonoids were identified in the methanol extract of *M*. *oleifera* [42]. Based on ultra-high-performance liquid chromatography (UHPLC) coupled with electrospray ionization quadrupole time-of-flight mass spectrometry (UHPLC-ESI-q TOF-MS), 14 flavonoids were identified in *M*. *oleifera* leaves collected in both South Africa and Namibia [43]. It is noteworthy that even in the presence of factors influencing the distribution of flavonoids, such as variations in environmental conditions as well as solvent system or methods of analysis, the content of these phytochemicals in *M*. *oleifera* leaves remains highly important. This further confirms the health-promoting effects of the plant leaves.

The use of a rat model of obesity that raised from the administration of HFD consumption allowed us to verify that obesity is associated with an increase in both food efficiency ratio level and body weight gain in rats. The suppression of body weight gain showed in the present study substantiated the antiobesity potential of MEML in HFD-fed rats. These findings are consistent with previous data which reported that *M*. *oleifera* leaf extract was efficient in the treatment of weight gain [44]. Several studies confirm an inverse relationship between the intake of dietary fiber, minerals, vitamins, phytochemicals, and antioxidants, and the development of obesity and its comorbidities [45, 46].

In order to determine whether MEML administration had an effect on circulating lipid levels, a comprehensive serum lipid profile assay was carried out. The HFD group revealed a significant increase in the rates of total cholesterol, triglyceride, and LDL-cholesterol in serum, compared to the control group. The supplementation of HFD-fed rats with MEML at 200 and 400 mg/kg doses significantly inhibited the levels of total cholesterol, triglycerides, and LDL-cholesterol and increased HDL-cholesterol level in the serum compared to the HFD-only group. This suggested that MEML consumption-induced alleviation of the hyperlipidemia and harmful cardiovascular effects exerted by HFD feeding.

Serum CK-MB, AST, and ALT are the enzyme biomarkers which allow monitoring of the heart structural integrity and damage and serve as clinical indicators in the diagnosis of heart complications [47]. In the presence of a damage stimulus, additional CK-MB, AST, and ALT are released into the bloodstream and enhance the serum enzyme level reflecting the degree of tissue injury. Our results indicated that CK-MB, AST, and ALT amounts increased significantly in the HDF-treated rats (*p* < 0.01) compared to the control group. However, it was found that administration of both low and high MEML doses in the HFD-treated group successfully alleviated these abnormalities. The evidence that *M*. *oleifera* leaf extract can influence cardiovascular disease risk parameters, when high-fat diet is consumed, could be attributed to the phytochemical content in MEML and its antioxidant potentials.

Oxidative stress supervenes as a result of instability between the levels of antioxidants and oxidants in favor of oxidants [48]. Numerous studies claimed that high-fat diet incites metabolic pattern leading to the generation of reactive oxygen species (ROS) implicated in the etiology of several health complications such as atherosclerosis, diabetes, cancer, cardiovascular disorders, and other chronic diseases [49, 50]. Glutathione peroxidase (GPx), catalase (CAT), and superoxide dismutase (SOD) are antioxidant enzymes basically included in the antioxidant protective capacity of biological systems against free radical attack [51].

Our study showed that diet-induced obesity markedly impaired the antioxidant status in heart tissues of HDF-treated rats by increasing the levels of TBARS and reducing the SOD, CAT, and GPx activities, compared to controls. Furthermore, the administration of HFD was found to decrease cardiac TSH levels. TSH, like other nonenzymatic antioxidants (including reduced glutathione, vitamins C and E, natural flavonoids, and other compounds), interacts synergistically with the enzymatic antioxidant system and often acts to neutralize or scavenge free radicals by donating electrons [52]. Oral administration of MEML in the diet of HFD-treated rats significantly reduced the lipid peroxidation levels and restored the antioxidant enzyme activities as compared with control rats. These findings strongly suggest that *M*. *oleifera* extract is highly effective at preventing heart tissue damage by reducing lipid peroxidation and keeping the ROS levels at acceptable cellular concentrations. These results are consistent with previously reported data [53]. On the other hand, the cardiac histoarchitecture of the HFD-treated rats exhibited myocardial fiber disorganization, inflammatory cell collections, and area of myocardial fibrosis. MEML supplementation also induced a noticeable improvement in the histological alterations in the HFD-fed rats and minimised cardiac injury. Our findings are in agreement with those reported by Zeng et al. [54] and Poudyal et al. [55] who proved degenerative cardiac changes in rats and mice, respectively, in the setting of obesity.

## 5. Conclusion

In summary, the abovementioned biochemical parameters and histological evidence indicated that the methanolic extract of *M*. *oleifera* leaves at 200 and 400 mg/kg bw played an effective role in the treatment of obesity and reduction of cardiometabolic abnormalities. This prominent therapeutic activity is mainly attributed to the considerable amounts of health-promoting nutrients and phenolic compounds in this plant. However, further investigation is necessary to isolate bioactive compounds of Moringa leaf extract to examine their mechanistic therapeutic role and to avoid their potential toxic effects.

## Figures and Tables

**Table 1:** Composition of experimental diets.

Composition (%)	Normal diet (ND)	High-fat diet (HFD)^∗^
Sucrose	2	4
Lard	2.5	34
Soybean oil	2.5	3
*α*-Corn starch	10	15
Maltodextrin	29.64	6
Casein	18.5	25.5
L-Cystine	0.26	0.36
Cellulose	6.65	6.65
Mineral mix	4	4
Calcium carbonate	0.2	0.24
Vitamin mix	1.00	1.00
Choline bitartrate	0.25	0.25
Total	100	100

^∗^ High-fat diet was modified from the AIN-76 dietary composition [19].

**Table 2:** Proximate composition and mineral content of *Moringa oleifera* leaves.

Components	Amount
Moisture (g/100 g DW)	6.82 ± 0.181
Crude proteins (g/100 g DW)	25.87 ± 0.340
Crude fats (g/100 g DW)	7.28 ± 0.173
Ash (g/100 g DW)	10.73 ± 0.021
Crude fibers (g/100 g DW)	7.98 ± 0.050
Carbohydrates (g/100 g DW)	41.32 ± 0.263
Sugar (g/100 g DW)	9.18 ± 0.012
Energy (kcal/100 g DW)	350 ± 0.178
Sodium (Na) (mg/100 g)	660.01 ± 0.151
Calcium (Ca) (mg/100 g)	1,942.47 ± 11.230
Magnesium (Mg) (mg/100 g)	464.21 ± 1.214
Potassium(K) (mg/100 g)	1,134.12 ± 0.463
Phosphorus (P) (mg/100 g)	103.89 ± 0.521
Iron (Fe) (mg/100 g)	34.56 ± 0.987
Zinc (Zn) (mg/100 g)	2.93 ± 0.064
Manganese (Mn) (mg/100 g)	4.72 ± 0.040

Values are means ± SD of three replicate determinations.

**Table 3:** Total phenolic, flavonoid, and tannin contents in *Moringa oleifera* methanol extract.

Parameters	Content
Total phenols (mg gallic acid equivalents/g extract)	247.53 ± 3.24
Flavonoids (mg rutin equivalents/g of extract)	34.13 ± 2.7
Tannins (mg catechin equivalents/g of extract)	10.5 ± 0.79

Values are means ± SD of three replicate determinations.

**Table 4:** Phenolic compounds identified in methanol extract of *Moringa oleifera* leaves by LC-ESI-MS.

No.	Compounds^∗^	*R*_*t*_ (min)	Molecular formula	Molecular mass	(M-H) (m/z)	Content (*μ*g/g extract)
1	Quinic acid	2,017	C_7_H_12_O_6_	192	191	16.739
2	Protocatchuic acid	6,485	C_7_H_6_O_4_	154	153	4.969
3	Epicatechin	16,120	*C_15_H_14_O_6_*	290	289	0.091
4	p-Coumaric acid	20,688	C_9_H_8_O_3_	164	163	0.888
5	*trans*-Ferrelic acid	22,729	C_10_H_10_O_4_	194	193	0.958
6	Rutin	23,545	C_27_H_30_O_16_	610	609	47.401
7	Hyperoside	24,253	C_21_H_20_O_12_	464	463	316.822
8	Naringin	25,817	C_27_H_32_O_14_	580	579	9.400
9	Quercetrin	26,138	C_21_H_20_O_11_	448	447	204.685
10	3,4-di-*O*-Caffeoylquinic acid	26,483	C_25_H_24_O_12_	516	515	1.342
11	Salviolonic acid	27,883	C_36_H_30_O_16_	718	717	2.330
12	Quercetin	31,570	C_15_H_10_O_7_	302	301	3.148
13	Kaempferol	31,639	C_15_H_10_O_6_	286	285	0.489
14	Apigenin	34,193	C_15_H_10_O_5_	270	269	0.126
15	Luteolin	34,596	C_15_H_10_O_6_	286	285	1.958
16	Cirsiliol	35,206	C_17_H_14_O_7_	330	329	17.757
17	Cirsilineol	38,132	C_18_H_16_O_7_	344	343	0.280
18	Acacetin	39,687	C_16_H_12_O_5_	284	283	0.483

^∗^Identification was verified using 31 authentic commercial standards.

**Table 5:** Effect of MEML on body weight gain, feed intake, feed efficiency ratio, serum lipid profiles, and cardiac marker enzymes in obese rats.

Parameters	C	HFD	MEML 200	MEML 400
Body weight gain (%)	98.04	162.5^∗^	145.33^++^	126.15^++^
Feed intake (g/week)	115.2 ± 3.12	122.51±2.7^∗∗^	119.82 ± 1.27^++^	119.09 ± 4.22^++^
Feed efficiency ratio (%)	4.8	8.19^∗∗^	7.31^++^	6.05^+^
Total cholesterol(mg/dL)	89.15 ± 2.16	95.76±11.09^∗∗^	93.26 ± 4.32^++^	92.03 ± 1.43^++^
Triglycerides (mg/dL)	218.69 ± 4.85	265.15±7.12^∗∗^	232.67 ± 2.94^++^	227.50 ± 3.13^++^
HDL-C (mg/dL)	63.98 ± 1.5	30.22±5.22^∗∗^	39.04 ± 2.45^++^	47.32 ± 5.08^++^
LDL-C (mg/dL)	44.65 ± 2.41	59.33±0.16^∗∗^	48.15 ± 0.96^++^	44.76 ± 2.20^++^
CK-MB (UI/L)	204.30 ± 10.28	392.64±8.12^∗∗^	311.55 ± 22.34^++^	226.03 ± 5.23^++^
AST (UI/L)	49.5 ± 9.18	98.85±7.08^∗∗^	77.56 ± 2.87^++^	60.45 ± 7.22^++^
ALT (UI/L)	50.04 ± 12.45	102.13±4.56^∗∗^	76.40 ± 13.08^++^	61.88 ± 11.13^++^

Values are expressed as mean ± SD (*n* = 6). ^∗^*p* < 0.05, ^∗∗^*p* < 0.01 versus the control group (C). ^+^*p* < 0.05, ^++^*p* < 0.01 versus the high-fat diet group (HFD).

**Table 6:** Lipid peroxidation levels (TBARS), nonenzymatic antioxidant levels (TSH), and enzymatic antioxidant activities (glutathione peroxidase, catalase, and superoxide dismutase) in the hearts of the different groups.

Parameters	C	HFD	MEML 200	MEML 400
TBARS (nmol/mg protein)	0.61 ± 0.34	1.31 ± 0.23^∗∗^	1.05 ± 0.49^++^	0.77 ± 0.3^++^
SOD (U/mg of protein)	10.01 ± 0.26	6.95 ± 0.08^∗^	7.82 ± 0.21^+^	8.97 ± 1.09^++^
CAT (*μ*mol/min/mg of protein)	14.89 ± 2.11	9.52 ± 1.2^∗∗^	11.75 ± 0.48^++^	13.01 ± 0.7^++^
GPx (*μ*mol GSH oxidized/min/mg of protein)	6.83 ± 0.65	4.01 ± 1.1^∗∗^	5.1 ± 1.8^++^	6.05 ± 0.04^++^
TSH (U/mg protein)	1.92 ± 0.1	0.51 ± 0.02^∗∗^	0.72 ± 0.33^++^	0.89 ± 0.1^++^

Values are expressed as mean ± SD (*n* = 6). ^∗^*p* < 0.05, ^∗∗^*p* < 0.01 versus the control group (C). ^+^*p* < 0.05, ^++^*p* < 0.01 versus the high-fat diet group (HFD). C: control rats; HFD: rats fed with high-fat diet; MEML 200: rats fed a high-fat diet and treated with methanolic extract of *M*. *oleifera* leaves (200 mg/kg); MEML 400: rats treated with methanolic extract of *M*. *oleifera* leaves (400 mg/kg) along with high-fat diet for 12 weeks.

**Table 7:** The Pearson correlation coefficients between the parameters.

	Feed intake	Body weight gain	Feed efficiency ratio
Total cholesterol	0.993	0.991	0.984
Triglycerides	0.895	0.881	0.886
HDL-C	-0.972	-0.986	-0.976
LDL-C	0.855	0.859	0.873
CK-MB	0.898	0.941	0.960
AST	0.940	0.966	0.977
ALT	0.935	0.970	0.983
TBARS	0.935	0.971	0.984
SOD	-0.963	-0.994	-0.999
CAT	-0.978	-0.985	-0.985
GPx	-0.958	-0.978	-0.984
TSH	-0.963	-0.949	-0.924

## Data Availability

All data generated or analysed during the current study are available from the corresponding author on reasonable request.

## Additional Points

*Novelty*. (i) High nutrients and phenolic compound diversity were assessed in the *M*. *oleifera* leaves. (ii) Methanolic extract of *M*. *oleifera* leaves exhibited a defensive role against obesity biomarkers and cardiac damages.
